# Effects of anthocyanin supplementation on blood lipid levels: a systematic review and meta-analysis

**DOI:** 10.3389/fnut.2023.1207751

**Published:** 2023-08-15

**Authors:** Hwan-Hee Jang, In-Guk Hwang, Young-Min Lee

**Affiliations:** ^1^Functional Food Division, National Institute of Agricultural Sciences, Rural Development Administration, Wanju, Republic of Korea; ^2^Department of Practical Science Education, Gyeongin National University of Education, Incheon, Republic of Korea

**Keywords:** metabolic syndrome, dyslipidemia, food supplementation, triglyceride, HDL cholesterol

## Abstract

**Introduction:**

Dyslipidemia is a major cardiovascular disease risk factor associated with increased mortality. The intake of plant food-derived bioactive compounds is associated with beneficial cardiovascular effects, including decreased blood lipid levels and cardiovascular risk. We aimed to evaluate the effects of anthocyanin intake on blood lipid levels by analyzing relevant randomized controlled trials.

**Methods:**

We searched the PubMed and Embase databases using the “Patient/Population, Intervention, Comparison, and Outcomes” format to determine whether anthocyanin supplementation intervention affected blood lipid levels compared with placebo supplementation in human participants.

**Results:**

A total of 41 studies with 2,788 participants were included in the meta-analysis. Anthocyanin supplementation significantly reduced triglyceride [standardized mean difference (SMD) = −0.10; 95% confidence interval [CI], −0.18, −0.01) and low-density lipoprotein-cholesterol (SMD = −0.16; 95% CI −0.26, −0.07) levels and increased high-density lipoprotein-cholesterol levels (SMD = 0.42; 95% CI 0.20, 0.65).

**Discussion:**

Anthocyanin supplementation significantly improved blood lipid component levels in the included studies. Larger, well-designed clinical trials are needed to further investigate the effects of anthocyanin intake on blood lipid levels and the safety of anthocyanin supplementation for treating dyslipidemia.

**Systematic review registration:**

https://www.crd.york.ac.uk/prospero/display_record.php?ID=CRD42021257087, identifier: CRD42021257087.

## 1. Introduction

Dyslipidemia, one of the key components of metabolic syndrome, is a major risk factor for cardiovascular disease (CVD) and increased mortality (1, 2). Globally, CVD is a leading cause of morbidity and mortality; hence, the priority of developing effective means to reduce CVD risk cannot be overstated. Lifestyle changes, such as eating a healthy diet and increasing physical activity, have been shown to reduce CVD risk. Various bioactive compounds derived from plant foods are also associated with beneficial cardiovascular effects and CVD risk reduction.

Water-soluble anthocyanin pigment supplementation, which is a secondary metabolite belonging to the flavonoids family, has antioxidant and antiinflammatory properties and many health benefits including protection against carcinoma and diabetes. The positive effects of anthocyanin supplementation on dyslipidemia have been reported in several clinical trials. However, although meta-analyses of randomized controlled trials have been conducted, the relationship between anthocyanin supplementation and dyslipidemia has not been consistently reviewed to date (3–5).

Cyanidin, a type of anthocyanin, is a pigment mainly found in red-skinned or red-fleshed fruits, including apples, hawthorn berries, bilberries, cranberries, chokeberries or aronia berries, and lingonberries (6). Numerous *in vitro* and animal studies have reported that cyanidin may modulate the lipid metabolism (7–9); however, systematic reviews and meta-analyses on the lipid profile improvement effect of cyanidin in human are lacking.

In the present study, we aimed to analyze the effects of anthocyanin supplementation on blood lipid levels by conducting a systematic review and meta-analysis of relevant randomized control trials.

## 2. Materials and methods

This systematic review and meta-analysis were reported in accordance with the Preferred Reporting Items for Systematic Reviews and Meta-Analyses (PRISMA) guidelines (Appendix 1) (10). The protocol was registered in PROSPERO (CRD42021257087).

### 2.1. Search strategy

The PubMed and Embase databases were searched from their inception to June 2023 using the search terms “Anthocyanin OR Cyanidin” and “Black food” to identify relevant articles. Only studies conducted in clinical settings and published in the English language were considered. Additional articles were identified *via* a manual search of the reference lists of the original articles, reviews, and meta-analyses. The duplicate results were eliminated using EndNote software, and the titles and abstracts were screened by two authors (H-HJ and Y-ML) using Rayyan QCRI online software (https://www.rayyan.ai). The relevant studies then underwent dual full-text screening.

### 2.2. Inclusion criteria

The meta-analysis was performed using the “Patient/Population, Intervention, Comparison, and Outcomes” format to determine whether an intervention with anthocyanin supplementation (I) had any effect on blood lipids (O) compared with placebo supplementation (C) among participants (P). The outcomes of interest were levels of triglyceride (TG), total cholesterol, low-density lipoprotein (LDL)-cholesterol, and high-density lipoprotein (HDL)-cholesterol. Parallel or crossover randomized control trials were included, whereas observational studies and review articles were excluded. The intervention duration and dose were at least 2 weeks and 10 mg/day, respectively. Two authors (H-HJ and Y-ML) independently reviewed data from all the studies that fulfilled the inclusion criteria, and any conflicts were consensually resolved.

### 2.3. Data extraction and risk of bias assessment

Two reviewers (H-HJ and Y-ML) independently extracted the following data from the included studies: authors, year of publication, study design, place, study population (age, number, proportion of women, and health status), and intervention (sources, dose, type, duration, and concentration of anthocyanin and cyanidin) (Table 1). Two reviewers (H-HJ and Y-ML) independently assessed the risk of bias using the Jadad scale (52). This scale considers randomization, blinding, and accountability of all patients. If all these items are regarded as appropriate, a score of 5 is assigned. The Jadad scale scores of ≥3 and <3 were considered to have a low and high risk of bias, respectively. Any disagreements were consensually resolved.

**Table 1:** Characteristics and findings of the studies included in the meta-analysis.

**Study (Ref.)**	**Design**	**Place**	**Participants (% of women)**	**Age (in years)**	**Duration (weeks)**	**Healthy status**	**Food sources**	**Intervention types**	**Anthocyanin (mg/day)**	**Cyanidin (mg/day)**	**Jadad scale**
Murkovic et al. (11)	PA	Austria	34 (41%)	29	2	Healthy	Elderberry	Capsule	120	120	3
Hansen et al. (12)	PA	Denmark	50 (56%)	53	4	Healthy	Red grape	Tablets	28.8 or 57.5	NI	4
Zern et al. (13)	CO	USA	44 (100%)	48	4	Healthy	Grape	Lyophilized powder	27.7	NI	1
Cerda et al. (14)	PA	Spain	30 (0%)	62	5	Patients with stable COPD	Pomegranates	Juice	190	NI	3
Karlsen et al. (15)	PA	USA	118 (51%)	61	3	Healthy	Bilberry and blackcurrant	Capsule	300	NI	1
Erlund et al. (16)	PA	Finland	71 (65%)	58	8	With CVD risk	Bilberry, lingonberry, blackcurrant, and strawberry	Mixed types (crushed, purée, and juice)	515	515	2
Curtis et al. (17)	PA	UK	50 (100%)	58	12	Healthy	Elderberry	Capsule	500	500	4
Qin et al. (18)	PA	China	120 (65%)	55	12	Healthy	Bilberry and blackcurrant	Capsule	320	105.6	4
Basu et al. (19)	PA	USA	48 (97%)	50	8	MetS	Blueberry	Lyophilized powder	742	NI	2
Stull et al. (20)	PA	USA	32 (84%)	51	6	Obese	Blueberry	Lyophilized powder	668	NI	4
Basu et al. (21)	PA	USA	36 (100%)	52	8	MetS	Cranberry	Juice	24.8	12.6	3
Dohadwala et al. (22)	CO	USA	44 (32%)	62	4	CAD	Cranberry	Juice	94	NI	4
Zhu et al. (23)	PA	China	146 (58%)	40–65	12	Dyslipidemia	Bilberry and blackcurrant	Capsule	320	105.6	3
Hassellund et al. (24)	CO	Norway	27 (0%)	41	4	Pre-hypertensive	Bilberry and blackcurrant	Capsule	640	211.2	5
Riso et al. (25)	CO	USA	18 (0%)	48	6	Healthy	Wild blueberry	Lyophilized powder	375	NI	5
Flammer et al. (26)	PA	USA	69 (45%)	50	16	With CVD risk	Cranberry	Juice	69.5	NI	3
Wright et al. (27)	PA	Australia	16 (0%)	53	4	Overweight and Obese	Purple carrot	Dried	118.5	118.5	4
Zhu et al. (28)	PA	China	146 (58%)	56	24	Dyslipidemia	Bilberry and blackcurrant	Capsule	320	105.6	4
Basu et al. (29)	PA	USA	60 (92%)	49	12	MetS	Strawberry	Freeze-dried	78 or 155	78 or 155	2
Lynn et al. (30)	PA	UK	43 (63%)	38	6	Healthy	Tart cherry	Juice	273.5	NI	3
Soltani et al. (31)	PA	Iran	50 (50%)	47	4	Dyslipidemia	Whortleberry	Capsule	90	90	5
Li et al. (32)	PA	China	58 (41%)	58	24	T2D	Bilberry and blackcurrant	Capsule	320	105.6	3
Novotny et al. (33)	PA	USA	56 (54%)	51	8	Healthy	Cranberry	Juice	20.6	NI	5
Soltani et al. (34)	PA	Iran	60 (35%)	50	6	T2D	Cornelian cherry	Capsule	600	NI	4
Stull et al. (35)	PA	USA	44 (64%)	57	6	MetS	Blueberry	Lyophilized powder	580.6	NI	5
Zhang et al. (36)	PA	China	74 (47%)	46	12	NAFLD	Bilberry and blackcurrant	Capsule	320	105.6	5
Lee et al. (37)	PA	Korea	63 (38%)	31	8	Overweight and Obese	Black soybean	Capsule	31.5	21.5	4
Zhang et al. (38)	PA	China	146 (58%)	56	24	Dyslipidemia	NI	Capsule (Polyphenols AS)	320	NI	3
Xie et al. (39)	PA	China	160 (66%)	61	12	Pre-diabetes	Bilberry and blackcurrant	Capsule	320	105.6	5
Yang et al. (40)	PA	USA	49 (51%)	35	12	Healthy	Aronia	Capsule	45.1	45.1	5
Hollands et al. (41)	CO	UK	38 (51%)	52	4	Healthy	Blood orange	Juice	50	NI	3
Kim et al. (42)	PA	USA	37 (70%)	44	12	MetS	Açaí berry	Juice	99.8	99.8	4
Bakuradze et al. (43)	PA	Germany	57 (0%)	24	24	Healthy	Red grape, lingonberry, blueberry, and aronia berry	Juice	205.9	75.4	2
Curtis et al. (44)	PA	UK	115 (32%)	63	9	MetS	Blueberry	Lyophilized powder	182 or 364	NI	5
Guo et al. (45)	PA	China	107 (67%)	25	2	Healthy	Bilberry and blackcurrant	capsule	20, 40, 80, 160, 320	6.6, 13.2, 26.4, 52.8, 105.6	5
Stote et al. (46)	PA	USA	52 (0%)	67	8	T2D	Blueberry	Lyophilized powder	261.8	NI	4
Chan et al. (47)	CO	China	20 (55%)	56	4	T2D	Bilberry	capsule	>350	NI	5
Sekikawa et al. (48)	PA	Japan	32 (50%)	37	6	Healthy	Bilberry	Capsule	43.2	NI	5
Xu et al. (49)	PA	China	176 (74%)	57	12	Dyslipidemia	Bilberry and blackcurrant	Capsule	40, 80, 320	13.2, 26.4, 105.6	5
Yang et al. (50)	PA	China	140 (67%)	61	12	prediabetes	Bilberry and black currant	Capsule (Biolink AS)	320	NI	5
Aboufarrang et al. (51)	CO	UK	52 (54%)	63	4	Dyslipidemia	Bilberry or black rice	Capsule	320	54 or 297.8	5

PA, parallel-arm; CO, crossover; COPD, chronic obstructive pulmonary disease; CVD, cardiovascular disease; NI, no information; CAD, coronary artery disease; T2D, type 2 diabetes; NAFLD, non-alcoholic fatty liver disease; MetS, metabolic syndrome.

### 2.4. Publication bias

We used Egger's regression test for funnel plot asymmetry to assess the potential publication bias of the included studies (53). *P*-values of <0.05 were considered significant.

### 2.5. Statistical analysis

We used the standardized mean difference (SMD) with the 95% confidence interval (CI) as effect size measures. In studies where the mean difference was not reported, the mean differences were calculated by subtracting the baseline mean from the post-intervention mean; the standard deviation (SD) differences were estimated using the following formula:


$${\text{SD}}_{\text{diff}}=\surd {\text{SD}}_{\text{B}}^{\text{2}}+{\text{SD}}_{\text{F}}^{\text{2}}-\text{2}\times \text{Corr}\times {\text{SD}}_{\text{B}}\times {\text{SD}}_{\text{F}},$$

where SD_B_ is the baseline SD and SD_F_ is the SD of the final measures in the study (54).

The correlation value was conservatively set at 0.5 to calculate the change in SD (55). Owing to the clinical heterogeneity of the studies, including differences in study design, doses, and intervention, a random-effects model was used for the meta-analysis of quantitative data. A forest plot was mapped to indicate the pooled SMD and 95% CI. Between-study heterogeneity was assessed by forest plot visualization. Subsequently, the *Q*-test and I^2^ statistic were used to quantitatively evaluate the statistical heterogeneity. In general, a *P-*value of <0.1 for Q statistics and an I^2^ > 50% indicate considerable heterogeneity (54). Sensitivity analysis was conducted to investigate the effect of each study on the pooled effect size if the I^2^ values were >50%. Furthermore, a subgroup analysis was performed to explore the possible source of heterogeneity for the following subsets: participants (number and healthy status), studies (design and area), or interventions (duration and dosage). All analyses were performed using R statistical software (Foundation for Statistical Computing, Vienna, Austria).

## 3. Results

### 3.1. Identification and selection of studies

**Figure 1:**
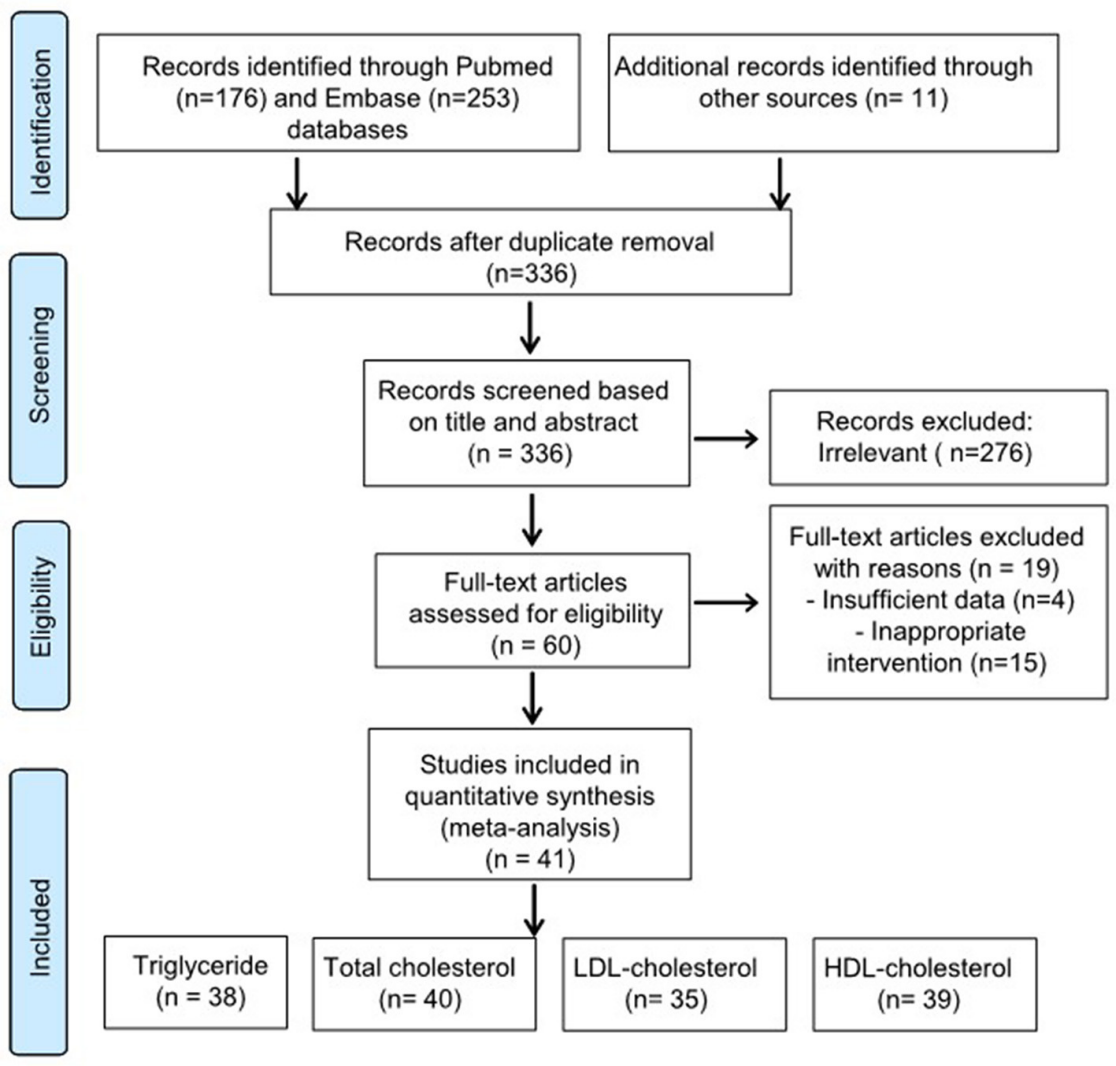
Preferred reporting items for systematic review and meta-analyses flowchart.

A total of 440 studies were initially identified: 429 from the database search and 11 from the manual search. A total of 104 duplicate studies were removed, and the titles and abstracts of 336 studies were screened by two authors. Subsequently, 276 studies were excluded. The remaining 60 studies underwent double full-text review. Subsequently, 19 studies were excluded: 4 were excluded because of insufficient data presentation (56–59) and 15 were excluded because of inappropriate interventions (60–74). Finally, 41 studies were included in the systematic review. The PRISMA flowchart for the selection process is presented in Figure 1.

### 3.2. Description of included studies

A total of 41 studies that enrolled 2,788 participants were included in the systematic review and meta-analysis. The characteristics of the included studies are summarized in Table 1. Among the 41 studies, 34 were parallel-arm studies (11, 12, 14–21, 23, 26–40, 42–46, 48–50), whereas 7 were crossover studies (13, 22, 24, 25, 41, 47, 51). Most studies included both sexes, three studies included only females (13, 17, 21) and six included only males (14, 24, 25, 27, 43, 46). All studies were conducted on adults, and the average age of the participants ranged from 24 to 67 years. Among the 41 studies, 14 were conducted in the United States (13, 15, 19–22, 25, 26, 29, 33, 35, 40, 42, 46), 11 in China (18, 23, 28, 32, 36, 38, 39, 45, 47, 49, 50), 5 in the UK (17, 30, 41, 44, 51), 2 in Iran (31, 34), and 1 each in Austria (11), Denmark (12), Spain (14), Finland (16), Norway (24), Australia (27), Korea (37), Germany (43), and Japan (48). Twenty-six studies were conducted in individuals with diseases such as chronic obstructive pulmonary disease (14), dyslipidemia (23, 28, 31, 38, 49), hypertension (24), obesity (20, 27, 37), type 2 diabetes mellitus (32, 34, 39, 46, 47, 50), CVDs (16, 22, 26), non-alcoholic fatty liver disease (36), and metabolic syndrome (19, 21, 29, 35, 42, 44), and 15 studies were conducted in healthy individuals (11–13, 15, 17, 18, 25, 30, 33, 40, 41, 43, 45, 48, 51). In total, 15 studies focused on an intervention with bilberry and black currant (15, 16, 18, 23, 24, 28, 32, 36, 39, 45, 47–51); 15 on elderberry (11, 17), blueberry (19, 20, 25, 35, 43, 46), cranberry (21, 22, 26, 33), whortleberry (31), açaí berry (42), or mixed fruits (43) supplementation; and 10 on grapes (12, 13), pomegranates (14), purple carrot (27), tart cherry (30), cornelian cherry (34), black soybean (37), aronia berries (40), strawberry (29), blood orange (41), and black rice (51) supplementation. One study (38) that reported on anthocyanin supplementation did not mention the food source. The duration of the interventions in the included studies varied (2–24 weeks). Among the 41 studies, 22 used supplements of concentrated anthocyanins in capsule (11, 15, 17, 18, 23, 24, 28, 31, 32, 34, 36–40, 45, 47–51) or tablet (12) form, 9 studies (14, 21, 22, 26, 30, 33, 41–43) used juice form supplements, and 9 other studies (13, 19, 20, 25, 27, 29, 35, 44, 46) used dried powder form supplements. One study (16) used mixed-type supplements, including juice and puree forms. The anthocyanin concentration ranged from a minimum of 20 mg/day to a maximum of 742 mg/day, with an average of 238.5 mg/day. Among the 41 studies, 21 presented intake levels of cyanidin with total anthocyanins. The total anthocyanin intake was 215.3 mg/day in 21 studies, which was marginally lower than the intake of 238.5 mg/day in all 41 studies. The cyanidin concentration of supplements ranged from a minimum of 6.6 mg/day to a maximum of 515 mg/day, with an average of 116.5 mg/day.

### 3.3. Potential sources of bias

The risk of bias assessments for individual studies are presented in Supplementary Table 1. Thirty-five of the included studies had a low risk of bias (Jadad score ≥ 3) and six had a high risk of bias (Jadad score <3). All 41 studies were randomized; however, only 20 appropriately described the method of randomization. Of the 33 studies that mentioned blinding, only 26 described the method of blinding. In 36 of the included 41 studies, the reasons for withdrawal or dropout of participants were described.

### 3.4. Outcomes

**Figure 2:**
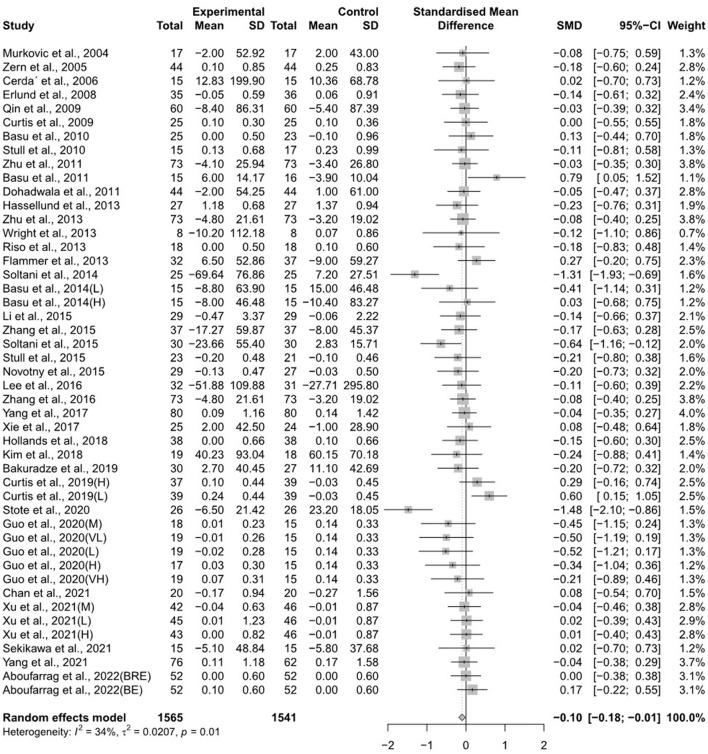
A forest plot of the change in the standardized mean differences (with 95% confidence intervals) of triglycerides in participants administered anthocyanin supplements compared with the control.

**Figure 3:**
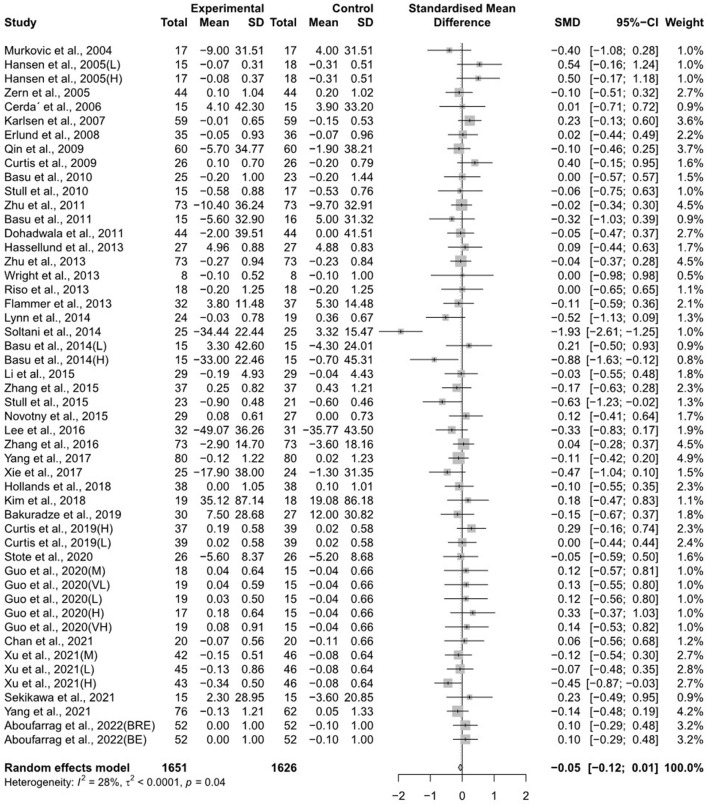
A forest plot of the change in the standardized mean differences (with 95% confidence intervals) of total cholesterol in participants administered anthocyanin supplements compared with the control.

**Figure 4:**
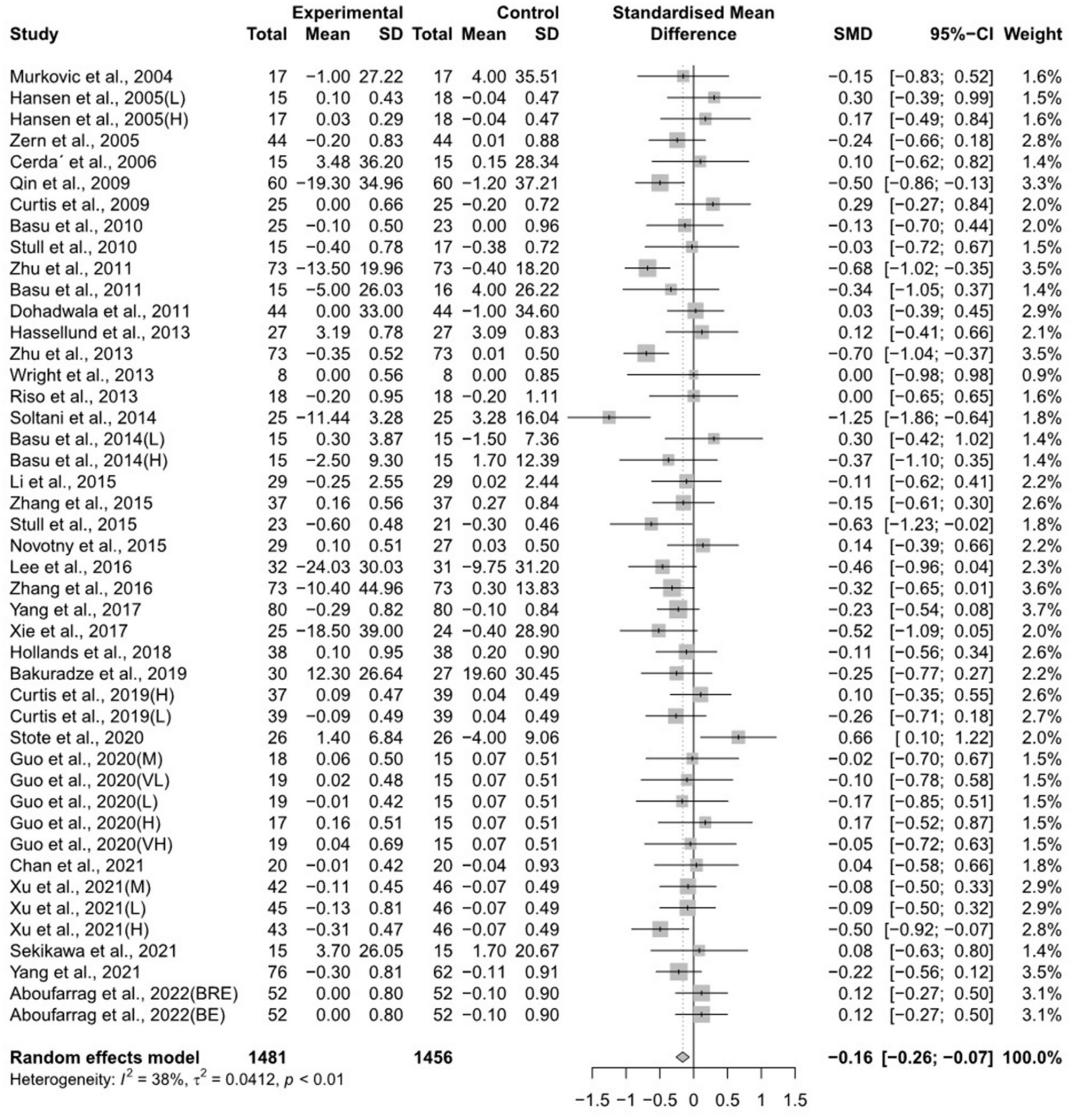
A forest plot of the change in the standardized mean differences (with 95% confidence intervals) of low-density lipoprotein-cholesterol in participants administered anthocyanin supplements compared with the control.

**Figure 5:**
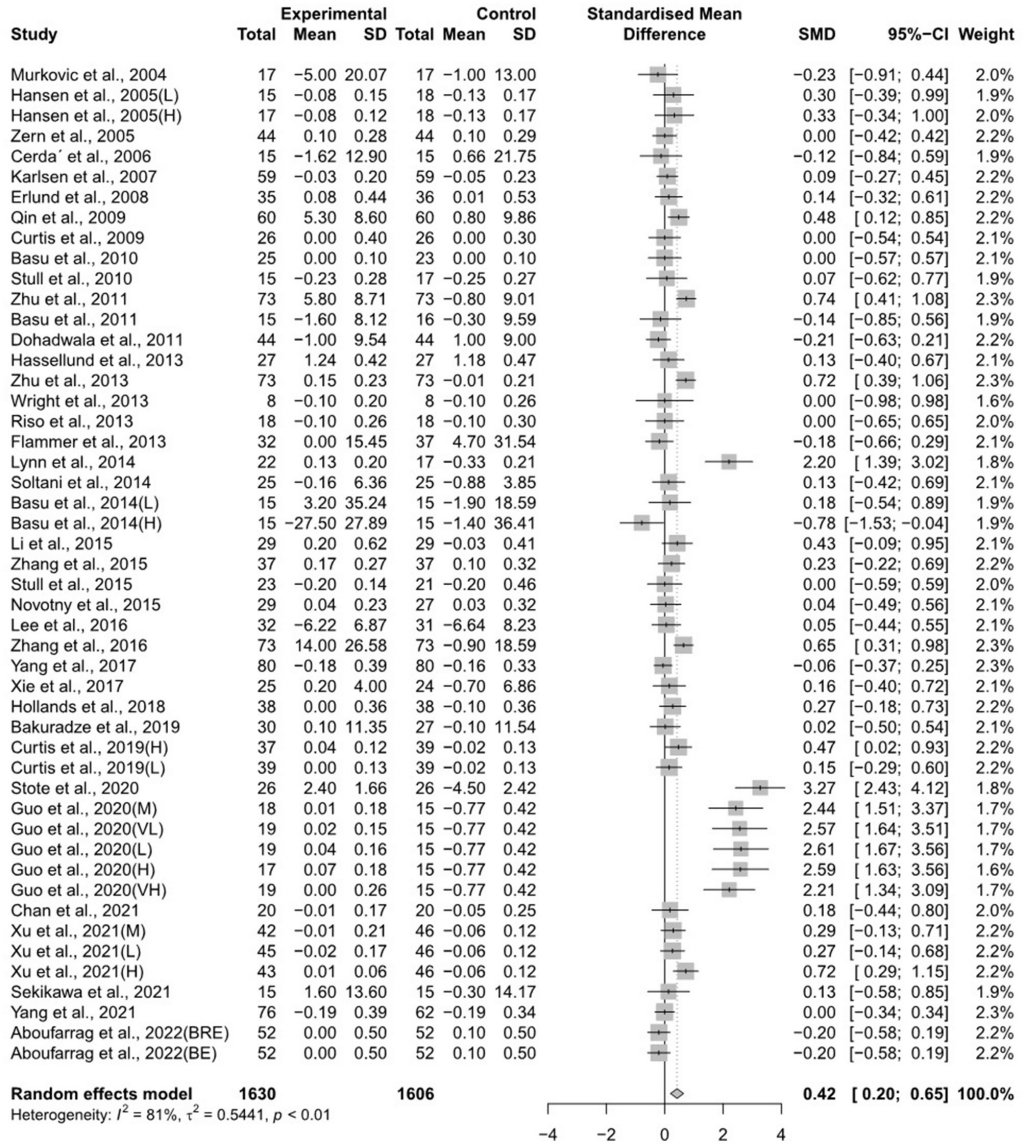
A forest plot of the change in the standardized mean differences (with 95% confidence intervals) of high-density lipoprotein-cholesterol in participants administered anthocyanin supplements compared with the control.

The forest plots for the overall random effects of anthocyanin-rich food supplementation on the levels of TG, total cholesterol, LDL-cholesterol, and HDL-cholesterol are illustrated in Figures 2–5. The overall pooled statistics revealed that the TG levels of participants (47 results from 38 studies) were significantly reduced (SMD = −0.10; 95% CI −0.18, −0.01), and a small degree of heterogeneity was observed in the analysis of TG (I^2^ = 34% and *P* = 0.01) (Figure 2). A total of 40 studies that included 50 effect sizes investigated the impact of anthocyanin supplementation on total cholesterol levels (Figure 3). The pooled statistics revealed that the SMD of total cholesterol was −0.05 (95% CI −0.12, 0.01), and a small degree of between-study heterogeneity was observed in the analysis (I^2^ = 28% and *P* = 0.04). Overall, 44 effect sizes from 35 studies were included in the analysis of the effect of anthocyanin supplements on LDL-cholesterol levels and revealed a significant effect (SMD = −0.16; 95% CI −0.26, −0.07) (Figure 4). The forest plot for the overall effect of anthocyanin supplements on HDL-cholesterol levels, which included the results of 39 studies, is presented in Figure 5. The SMD of the overall pooled HDL-cholesterol levels was 0.42 (95% CI 0.20, 0.65), which was a considerable increase. Statistical heterogeneity was observed in the analysis of LDL-cholesterol (I^2^ = 38% and *P* = 0.01) and HDL-cholesterol (I^2^ = 81% and *P* < 0.01) levels. Sensitivity analysis of the effect of anthocyanin supplementation on HDL-cholesterol levels revealed that the removal of any study did not alter the significance of the pooled effect size (Supplementary Figure 1). Note that, for the results of the meta-analysis of the 21 studies that included cyanidin intake, the effect on blood lipids had a greater impact than the results of all 41 studies (Supplementary Figures 2–5); moreover, cyanidin significantly reduced TG (SMD = −0.10; 95% CI −0.19, −0.01) and LDL-cholesterol (SMD = −0.23; 95% CI −0.37, −0.10) levels and increased HDL-cholesterol (SMD = 0.50; 95% CI 0.18, 0.82) levels.

### 3.5. Subgroup analyses

Furthermore, we performed subgroup analyses to explore the possible source of heterogeneity among the studies (Table 2). The effect of anthocyanin supplementation on HDL-cholesterol was significantly greater in the subgroup with a normal cholesterol level <200 mg/dL than in the subgroup with a higher cholesterol level ≥ 200 mg/dL at the baseline (*P* = 0.020). However, heterogeneity (I^2^) was 51% in hypercholesterolemic participants (total cholesterol ≥ 200) and lower than 91% in normal-level participants. The effect on HDL-cholesterol exhibited a higher increase in the subgroup with an average age <40 years (SMD = 1.30; 95% CI 0.54, 2.05) than in the subgroup with an average age ≥40 years (SMD = 0.21; 95% CI 0.07, 0.35). In particular, the effect of anthocyanin supplementation on HDL-cholesterol was significantly higher in studies with a low risk of bias than in the groups with a high risk of bias (*P* = 0.002). Subgroup analysis among study design, anthocyanin dosage, and risk of bias indicated decreased heterogeneity within subgroups. However, substantial unexplained heterogeneity was observed between these subgroups.

**Table 2:** Subgroup analysis for the effect of anthocyanin on high-density lipoprotein-cholesterol.

**Subgroup**	**Effect size**		**I^2^ ^2^ (%)**	***P*-value^3^**
	**K** ^1^	**(95% CI)**		
**Overall**	49	0.42 (0.20, 0.65)	81	
**Study design**				
Parallel study	41	0.52 (0.25, 0.79)	83	<0.001
Crossover study	8	−0.04 (−0.20, 0.12)	0	
**Study duration**				
<8 weeks	24	0.59 (0.18, 0.99)	85	0.220
≥8 weeks	25	0.30 (0.08, 0.51)	75	
**Cholesterol level** ^4^				
Normal (<200 mg/dL)	18	1.00 (0.42, 1.57)	91	0.020
Higher (≥ 200 mg/dL)	30	0.19 (0.06, 0.31)	51	
**Study area**				
East	20	0.80 (0.41, 1.19)	85	0.007
West	29	0.17 (−0.06, 0.40)	72	
**Participant's age**				
<40 years old	11	1.30 (0.54, 2.05)	91	0.007
≥40 years old	38	0.21 (0.07, 0.35)	68	
**No. of participants**				
<50	19	0.77 (0.27, 1.27)	86	0.052
≥50	30	0.24 (0.06, 0.42)	75	
**Anthocyanin dosage**				
<50 mg/day	10	0.54 (−0.06, 1.14)	83	0.099
≥50 and <350 mg/day	30	0.50 (0.18, 0.81)	85	
≥350 mg/day	9	0.14 (−0.05, 0.32)	0	
**Formula**				
Low processing	20	0.15 (0.02, 0.27)	79	0.194
High processing	29	0.54 (0.26, 0.83)	81	
**Risk of bias**				
Low risk	42	0.51 (0.25, 0.77)	83	0.002
High risk	7	0.01(−0.17, 0.20)	0	

^1^Number of studies combined. ^2^Overall test for heterogeneity within subgroups by the random effects model. ^3^Test for subgroup difference by random effect model. ^4^Excluding one study in which total cholesterol level was not presented at the baseline.

In addition, the difference in the effect of anthocyanin supplementation on lipid improvement was compared by dividing healthy participants and participants with dyslipidemia into subgroups (Supplementary Table 2). As a result of performing subgroup analysis according to dyslipidemia status, there was no significant difference between the two groups in TG and TC levels. However, the effect of anthocyanin supplementation on LDL-cholesterol (*P* = 0.027) and HDL-cholesterol (*P* = 0.020) was significantly different between the two groups. In particular, LDL-cholesterol showed a significant reduction only in dyslipidemia participants.

### 3.6. Publication bias

Publication bias was observed according to the results of Egger's test and demonstrated significant bias for LDL-cholesterol (*P* = 0.0253) and HDL-cholesterol (*P* = 0.0087) levels. Despite signs of publication bias using Egger's test, the Duval and Tweedie trim and fill method (75) revealed that the adjusted estimate remained significant (Supplementary Table 3 and Supplementary Figure 6). LDL-cholesterol (SMD = −0.13; 95% CI −0.21, −0.05) on anthocyanin supplementation still showed a significant reduction effect with zero heterogeneity (I^2^ = 0%) except for four outlier studies (23, 28, 31, 46). Excluding 10 outlier studies (29, 30, 45, 46, 51), the effect size of HDL-cholesterol decreased compared with the overall study results but showed low heterogeneity (I^2^= 31%) and a significantly increasing effect (SMD = 0.20; 95% CI 0.10, 0.30). There was no demonstrable publication bias for TG (*P* = 0.0697) and total cholesterol (*P* = 0.5943).

### 3.7. Adverse events

Among the 41 studies, 37 studies for anthocyanin supplements reported no serious adverse events leading to withdrawal. In four studies (19, 24, 40, 44), adverse events leading to withdrawal were reported in the anthocyanin supplement groups. In total, 6 studies (24, 37, 40, 47, 49, 50) reported mild adverse events, including dark stools, headache, insomnia, and diarrhea, and 17 studies (12–16, 20–22, 25–27, 33, 35, 39, 42, 43, 51) did not report any adverse events. The distribution of adverse events in the treatment groups and placebo is presented in Supplementary Table 4.

## 4. Discussion

The present study aimed to investigate the effects of anthocyanin supplements on blood lipid levels by focusing on the results of randomized controlled trials. A total of 41 studies that enrolled 2,788 participants were included in the meta-analysis, which revealed that anthocyanin supplements had significantly improved blood lipid levels; they reduced TG and LDL-cholesterol levels and increased HDL-cholesterol levels.

A meta-analysis of 12–13 randomized controlled trials reported that anthocyanin supplements did not significantly improve blood lipid levels; however, in the subgroup analyses, decreased total cholesterol and LDL-cholesterol levels were observed when anthocyanin supplementation exceeded 300 mg/day (3). Another meta-analysis of 27 trials indicated that anthocyanin supplements were associated with decreased total cholesterol and LDL-cholesterol levels and marginally increased HDL-cholesterol levels in both healthy subjects and in those with cardiometabolic disease; however, no significant effects were observed on TG levels (4). Shah and Shah (5) reported that anthocyanin supplements significantly reduced TG and LDL-cholesterol levels and increased HDL-cholesterol levels in both the healthy and patient populations, with no significant effect on total cholesterol levels through a meta-analysis of 9–13 randomized controlled trials. The present meta-analysis included 41 studies (2,788 participants) with inconsiderable heterogeneity and publication bias. Therefore, our results are expected to provide more reliable and integrative insight into the effect of anthocyanin supplements on blood lipid levels in both healthy and patient populations.

Several studies have elucidated the mechanisms responsible for the beneficial effects of anthocyanins on blood lipid levels. Anthocyanin reportedly increases cholesterol efflux from macrophages, contributing to reverse cholesterol transport (18, 76, 77). Moreover, it decreases the mass and activity of plasma cholesteryl ester transfer protein, which is associated with increased efficiency of reverse cholesterol transport (18). Anthocyanins can activate AMP-activated protein kinase, which inhibits cholesterol and TG synthesis by HMG-CoA and acetyl-CoA carboxylase inhibition, respectively (78). Furthermore, anthocyanin dose-dependently reduces the micellar solubility of cholesterol and exhibits a significant reduction in cholesterol uptake in Caco-2 cells (79–81). It reportedly increased the excretion of fecal neutral and acidic sterols in experimental animals fed a cholesterol-enriched diet (82, 83).

In the present study, anthocyanin supplements did not have a significant effect on total cholesterol levels. This was consistent with the report of Shah and Shah (5). However, anthocyanin supplementation led to significant improvements in the lipid profiles (total cholesterol, TG, HDL-cholesterol, and LDL-cholesterol) of patients with dyslipidemia (84). In addition, Daneshzad (3) showed that anthocyanin supplementation had significant effects on total cholesterol for more than 300 mg/day for more than 12 weeks. However, our subgroup analysis did not indicate significant differences between subgroups according to cholesterol levels (≥ 200 mg/dL or <200 mg/dL) or dosage or duration (data not shown). Meanwhile, the total cholesterol level did not predict the risk of CVD and coronary heart disease compared with TG, HDL-cholesterol, and lipid ratios (85, 86). Further studies are needed to identify the protective effects of anthocyanins on increased morbidity or mortality using lipid ratios such as the total cholesterol:HDL-cholesterol and TG:HDL-cholesterol ratio.

CVD can be prevented by appropriately addressing the major risk factors such as dyslipidemia, hypertension, oxidative stress, and inflammatory stress (87). The present study revealed that anthocyanins could help reduce CVD risk by decreasing blood TG and LDL-cholesterol levels and increasing HDL-cholesterol levels. Several studies have reported that anthocyanins may have a significant blood-pressure-lowering activity (88). Furthermore, anthocyanins have potent antioxidant and antiinflammatory effects (89). In addition, anthocyanin supplementation improved vascular function, which is a strong predictor for CVD (90). Thus, increased intake of anthocyanin-rich foods may effectively reduce CVD risk.

Among the included studies, 21 presented intake levels of cyanidin. The average of total anthocyanin intake was 215.3 mg/day in those studies, and the cyanidin concentration was 116.5 mg/day (6.6~515.0 mg/day). The effect of cyanidin on blood lipids had a greater impact than the results of total anthocyanin. One recent study (51) failed to compare the effect of the two anthocyanin types (cyanidin-type vs. delphinidin-type) on blood lipid levels. Further study is needed to identify and clarify the mechanism of anthocyanin's structure on each bioactivity.

The strengths of the present study are the inclusion of all clinical trials that investigated the effects of anthocyanin on blood lipid levels. However, this study has some limitations. First, only articles in the English language were included in the meta-analysis, which raised concerns regarding the identification and selection of relevant studies. Second, significant between-study heterogeneity unexplained by differences in the methods of anthocyanin intervention, study design, and study population was observed.

In conclusion, we evaluated the effects of anthocyanin supplementation on blood lipid levels *via* a systematic review and meta-analysis of randomized controlled trials. Our results revealed that anthocyanin supplementation had a significant effect on TG, LDL-cholesterol, and HDL-cholesterol levels. Larger, well-designed clinical trials are needed to investigate the efficacy and safety of anthocyanin supplementation for the treatment of dyslipidemia.

## Data availability statement

The original contributions presented in the study are included in the article/Supplementary material, further inquiries can be directed to the corresponding author.

## Author contributions

H-HJ and Y-ML: conceptualization, data curation, and writing—original draft preparation. Y-ML and I-GH: writing—reviewing and editing. All authors contributed to the article and approved the submitted version.
