# Supplementary material for: Effects of anthocyanin supplementation on blood lipid levels: a systematic review and meta-analysis
Source: Front Nutr. 2023 Aug 15;10:1207751. doi: 10.3389/fnut.2023.1207751 (PMC10463756; doi:10.3389/fnut.2023.1207751)
Supplement: Supplementary file 1 [file Data_Sheet_1.pdf]

Supplementary Material

Effects of Anthocyanin Supplementation on Blood Lipid Levels: A Systematic Review and Meta-Analysis

Hwan-Hee Jang, In-Guk Hwang, Young-Min Lee*

*** Correspondence:** Young-Min Lee: ymlee@ginue.ac.kr

1 Supplementary Tables

Supplementary Table 1. Quality assessment criteria used to assess the final 41 studies included in the systematic review

Study	Randomization	Method of randomization described and appropriate	Blinding mentioned	Method of blinding described and appropriate	Withdrawal and dropout of participants described	Total
Murkovic et al. 2004	1	0	1	0	1	3
Hansen et al. 2005	1	0	1	1	1	4
Zern et al. 2005	1	0	0	0	0	1
Cerda´ et al. 2006	1	0	1	1	0	3
Karlsen et al. 2007	1	0	0	0	0	1
Erlund et al. 2008	1	0	0	0	1	2
Curtis et al. 2009	1	0	1	1	1	4
Qin et al. 2009	1	0	1	1	1	4
Basu et al. 2010	1	0	0	0	1	2
Stull et al. 2010	1	0	1	1	1	4
Basu et al. 2011	1	0	1	0	1	3
Dohadwala et al. 2011	1	0	1	1	1	4

Zhu et al. 2011	1	0	1	0	1	3
Hassellund et al. 2013	1	1	1	1	1	5
Riso et al. 2013	1	1	1	1	1	5
Flammer et al. 2013	1	0	1	1	0	3
Wright et al. 2013	1	0	1	1	1	4
Zhu et al. 2013	1	1	1	0	1	4
Basu et al. 2014	1	0	0	0	1	2
Lynn et al. 2014	1	1	0	0	1	3
Soltani et al. 2014	1	1	1	1	1	5
Li et al., 2015	1	0	1	1	0	3
Novotny et al. 2015	1	1	1	1	1	5
Soltani et al. 2015	1	0	1	1	1	4
Stull et al. 2015	1	1	1	1	1	5
Zhang et al. 2015	1	1	1	1	1	5
Lee et al. 2016	1	1	1	0	1	4
Zhang et al. 2016	1	0	1	0	1	3
Xie et al. 2017	1	1	1	1	1	5
Yang et al. 2017b	1	1	1	1	1	5
Hollands et al. 2018	1	1	0	0	1	3
Kim et al. 2018	1	0	1	1	1	4
Bakuradze et al. 2019	1	0	0	0	1	2
Curtis et al. 2019	1	1	1	1	1	5
Guo et al. 2020	1	1	1	1	1	5
Stote et al. 2020	1	1	1	0	1	4
Chan et al. 2021	1	1	1	1	1	5
Sekikawa et al. 2021	1	1	1	1	1	5
Xu et al. 2021	1	1	1	1	1	5
Yang et al. 2021	1	1	1	1	1	5
Aboufarrag et al. 2022	1	1	1	1	1	5

Supplementary Table 2. Subgroup analysis for the effect of anthocyanin supplementation on blood lipids.

Subgroup	Triglyceride (n)	Triglyceride Effect size (95% CI)	I^{2} [a] (%)	Total-cholesterol (n)	Total-cholesterol Effect size (95% CI)	I^2 (%)	LDL-cholesterol (n)	LDL-cholesterol Effect size (95% CI)	I^2 (%)	HDL-cholesterol (n)	HDL-cholesterol Effect size (95% CI)	I (%
**Overall**	47	-0.10 (-0.18, -0.01)	34	50	-0.06 (-0.12, 0.01)	28	45	-0.16 (-0.26, -0.07)	38	49	0.42 (0.20, 0.65)	8
**Dyslipidemia[b]**												
Normal	18	-0.16 (-0.36, 0.05)	56	19	0.19 (-0.11, 0.15)	0	16	0.01 (-0.13, 0.15)	0	18	1.00 (0.42, 1.57)	9
higher	27	-0.09 (-0.15, 0.02)	0	30	-0.10 (-0.22, 0.01)	49	28	-0.25 (-0.37, -0.12)	47	30	0.19 (0.06, 0.31)	5
p-value[c]		0.657			0.379			0.027			0.020	

a. Overall test for heterogeneity within subgroups by random effect model.

b. Normal, baseline total cholesterol < 200 mg/dl; Higher, baseline total cholesterol ≥200 mg/dl; Excluding one or two study that total cholesterol level was not presented at baseline.

c. Test for subgroup difference by random effect model.

Supplementary Table 3. Results after adjustment for funnel plot asymmetry using the trim and fill method

	LDL-cholesterol			HDL-cholesterol		
	k [1]	Effect size (95% CI)	I^2 (%) [2]	k	Effect size (95% CI)	I^2 (%)
Overall	45	-0.16 (-0.26, -0.07)	38	49	0.42 (0.20, 0.65)	81
Trim and fill	59	-0.30 (-0.40, -0.20)	55	49	0.42 (0.20, 0.65)	81
Trim and fill with outliers removed	53	-0.23 (-0.30, -0.15)	14	52	0.33 (0.23, 0.43)	51
Outliers removed	41	-0.13 (-0.21, -0.05)	0	39	0.20 (0.10, 0.30)	31

[1]Number of studies combined

[2]Overall test for heterogeneity within subgroups by random effect model

Supplementary Table 4. Distribution of adverse events

Study	Reported adverse events (treatment group)	Reported adverse events (placebo)
Murkovic et al. 2004	No	No
Hansen et al. 2005	NI	NI
Zern et al. 2005	NI	NI
Cerda´ et al. 2006	NI	NI
Karlsen et al. 2007	NI	NI
Erlund et al. 2008	NI	NI
Curtis et al. 2009	No	Anal irritation (1)[1], recurrence of previously undisclosed condition (1)[1], lump found in routine breast scan (1)[1]
Qin et al. 2009	No	No
Basu et al. 2010	Nausea, vomiting, constipation, and diarrhea (9)[1]	No
Stull et al. 2010	NI	NI
Basu et al. 2011	NI	NI
Dohadwala et al. 2011	NI	NI
Zhu et al. 2011	No	No
Hassellund et al. 2013	Diarrhea (1)[1] Minor headache (3), dark stools (2), nausea (1)	No
Riso et al. 2013	NI	NI
Flammer et al. 2013	NI	NI
Wright et al. 2013	NI	NI
Zhu et al. 2013	No	No
Basu et al. 2014	No	No
Lynn et al. 2014	No	No
Soltani et al. 2014	No	No
Li et al., 2015	No	No
Novotny et al. 2015	NI	NI
Soltani et al. 2015	No	No
Stull et al. 2015	NI	NI
Zhang et al. 2015	No	No
Lee et al. 2016	Colds (2), headaches (2), bruising (1), menstrual pain (1), enteritis (1), ulitis (1)	Colds (1), diarrhea (1), muscular pain (1), Otitis (1), laryngitis (1), fatigue (1), xerophthalmia (1), rash (1), conjunctivitis (1), possible hepatosis (1)
Zhang et al. 2016	No	No
Xie et al. 2017	NI	NI
Yang et al. 2017b	Treatment group (7), placebo (3): dark stool (5), insomnia (1),	

Study	Reported adverse events (treatment group)	Reported adverse events (placebo)
	abdominal pain (1), diarrhea (1), dizziness (1), skin rash (1), withdrawal (4)[1]: treatment (1) placebo (3)	
Hollands et al. 2018	No	No
Kim et al. 2018	NI	NI
Bakuradze et al. 2019	NI	NI
Curtis et al. 2019	serious adverse events (1)[1]	No
Guo et al. 2020	No	No
Stote et al. 2020	No	No
Chan et al. 2021	Dark green stool (50%), poor sleeping, mild headache	No
Sekikawa et al. 2021	No	No
Xu et al. 2021	Dark stool (9), insomnia (1), diarrhea (1), dizziness (2), skin rash (1)	Dark stool (1), insomnia (1), abdominal (1), dizziness (1)
Yang et al. 2021	Dizziness (1), insomnia (1), dark stool (5)	Diarrhea (1)[1], abdominal pain (1), skin rash (1)[1]
Aboufarrag et al. 2022	No	No

[1]Withdrawal
