# Supplementary material for: Effects of anthocyanin supplementation on blood lipid levels: a systematic review and meta-analysis
Source: Front Nutr. 2023 Aug 15;10:1207751. doi: 10.3389/fnut.2023.1207751 (PMC10463756; doi:10.3389/fnut.2023.1207751)
Supplement: Supplementary file 2 [file Data_Sheet_2.pdf]

Supplementary Material

Effects of Anthocyanin Supplementation on Blood Lipid Levels: A Systematic Review and Meta-Analysis

Hwan-Hee Jang, In-Guk Hwang, Young-Min Lee*

*** Correspondence:** Young-Min Lee: ymlee@ginue.ac.kr

1. **Supplementary Figures**

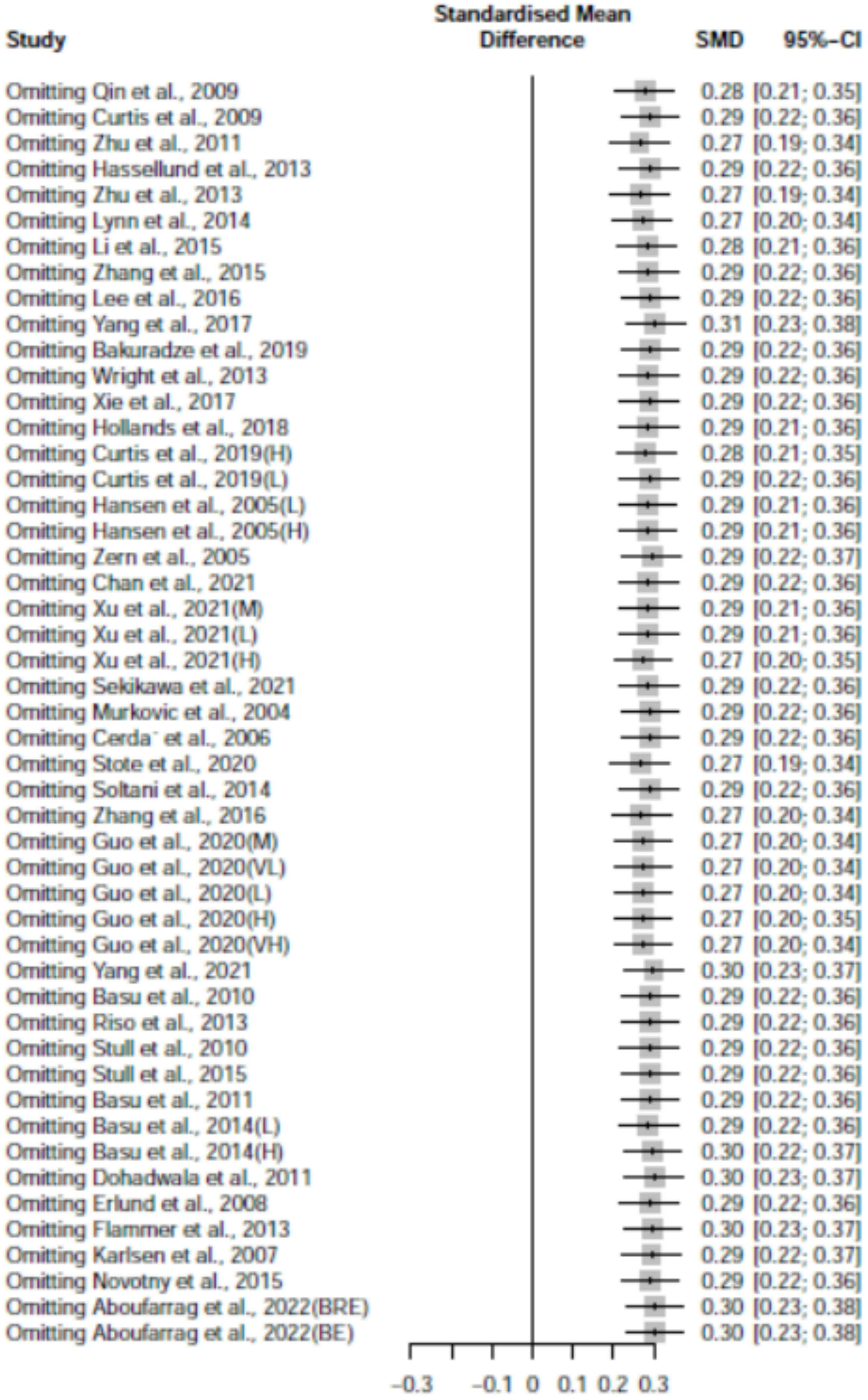

Supplementary Figure 1. Sensitivity analysis: high-density lipoprotein-cholesterol

Study	Experimental Total	Experimental Mean	Experimental SD	Control Total	Control Mean	Control SD	SMD	95%-CI	Weight
Murkovic et al., 2004	17	−2.00	52.92	17	2.00	43.00	−0.08	[−0.75; 0.59]	1.8%
Erlund et al., 2008	35	−0.05	0.59	36	0.06	0.91	−0.14	[−0.61; 0.32]	3.7%
Qin et al., 2009	60	−8.40	86.31	60	−5.40	87.39	−0.03	[−0.39; 0.32]	6.3%
Curtis et al., 2009	25	0.10	0.30	25	0.10	0.36	0.00	[−0.55; 0.55]	2.6%
Zhu et al., 2011	73	−4.10	25.94	73	−3.40	26.80	−0.03	[−0.35; 0.30]	7.7%
Basu et al., 2011	15	6.00	14.17	16	−3.90	10.04	0.79	[0.05; 1.52]	1.5%
Hassellund et al., 2013	27	1.18	0.68	27	1.37	0.94	−0.23	[−0.76; 0.31]	2.8%
Zhu et al., 2013	73	−4.80	21.61	73	−3.20	19.02	−0.08	[−0.40; 0.25]	7.7%
Wright et al., 2013	8	−10.20	112.18	8	0.07	0.86	−0.12	[−1.10; 0.86]	0.8%
Soltani et al., 2014	25	−69.64	76.86	25	7.20	27.51	−1.31	[−1.93; −0.69]	2.1%
Basu et al., 2014(L)	15	−8.80	63.90	15	15.00	46.48	−0.41	[−1.14; 0.31]	1.5%
Basu et al., 2014(H)	15	−8.00	46.48	15	−10.40	83.27	0.03	[−0.68; 0.75]	1.6%
Li et al., 2015	29	−0.47	3.37	29	−0.06	2.22	−0.14	[−0.66; 0.37]	3.0%
Zhang et al., 2015	37	−17.27	59.87	37	−8.00	45.37	−0.17	[−0.63; 0.28]	3.9%
Lee et al., 2016	32	−51.88	109.88	31	−27.71	295.80	−0.11	[−0.60; 0.39]	3.3%
Yang et al., 2017	80	0.09	1.16	80	0.14	1.42	−0.04	[−0.35; 0.27]	8.4%
Xie et al., 2017	25	2.00	42.50	24	−1.00	28.90	0.08	[−0.48; 0.64]	2.6%
Kim et al., 2018	19	40.23	93.04	18	60.15	70.18	−0.24	[−0.88; 0.41]	1.9%
Bakuradze et al., 2019	30	2.70	40.45	27	11.10	42.69	−0.20	[−0.72; 0.32]	3.0%
Guo et al., 2020(M)	18	0.01	0.23	15	0.14	0.33	−0.45	[−1.15; 0.24]	1.7%
Guo et al., 2020(VL)	19	−0.01	0.26	15	0.14	0.33	−0.50	[−1.19; 0.19]	1.7%
Guo et al., 2020(L)	19	−0.02	0.28	15	0.14	0.33	−0.52	[−1.21; 0.17]	1.7%
Guo et al., 2020(H)	17	0.03	0.30	15	0.14	0.33	−0.34	[−1.04; 0.36]	1.7%
Guo et al., 2020(VH)	19	0.07	0.31	15	0.14	0.33	−0.21	[−0.89; 0.46]	1.8%
Xu et al., 2021(M)	42	−0.04	0.63	46	−0.01	0.87	−0.04	[−0.46; 0.38]	4.6%
Xu et al., 2021(L)	45	0.01	1.23	46	−0.01	0.87	0.02	[−0.39; 0.43]	4.8%
Xu et al., 2021(H)	43	0.00	0.82	46	−0.01	0.87	0.01	[−0.40; 0.43]	4.7%
Aboufarrag et al., 2022(BRE)	52	0.00	0.60	52	0.00	0.60	0.00	[−0.38; 0.38]	5.5%
Aboufarrag et al., 2022(BE)	52	0.10	0.60	52	0.00	0.60	0.17	[−0.22; 0.55]	5.5%
**Random effects model**	**966**			**953**			**−0.10**	**[−0.19; −0.01]**	**100.0%**

Heterogeneity: $I^2 = 7\%$, $\tau^2 < 0.0001$, $p = 0.36$

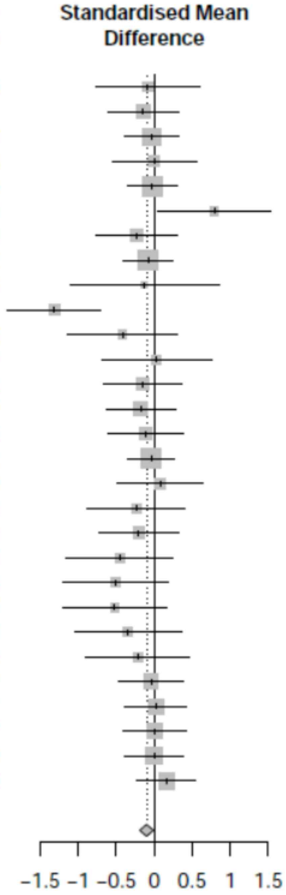

Supplementary Figure 2. Forest plot: Triglycerides

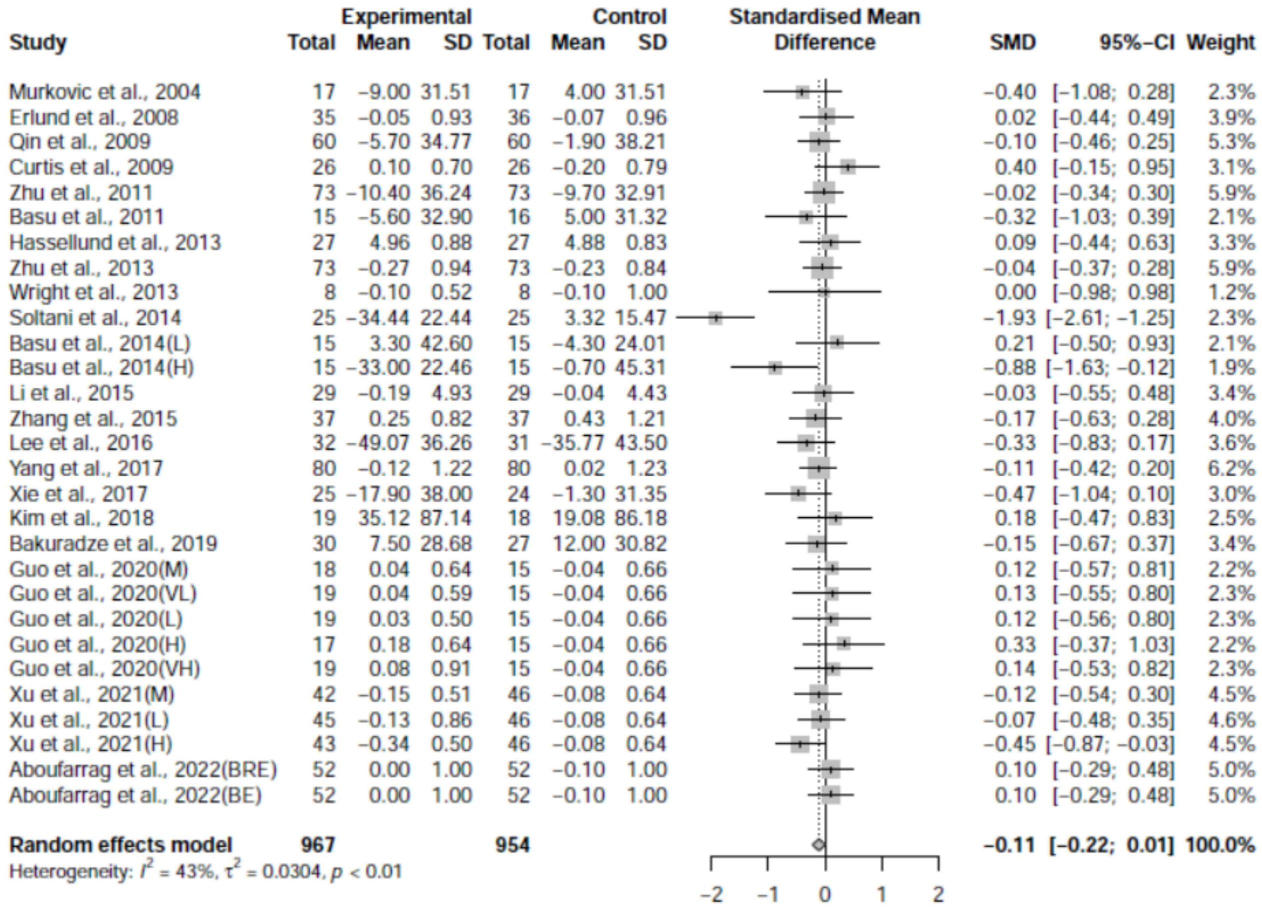

Supplementary Figure 3. Forest plot: total cholesterol

Study	Experimental Total	Experimental Mean	Experimental SD	Control Total	Control Mean	Control SD	SMD	95%-CI	Weight
Murkovic et al., 2004	17	−1.00	27.22	17	4.00	35.51	−0.15	[−0.83; 0.52]	2.7%
Qin et al., 2009	60	−19.30	34.96	60	−1.20	37.21	−0.50	[−0.86; −0.13]	5.2%
Curtis et al., 2009	25	0.00	0.66	25	−0.20	0.72	0.29	[−0.27; 0.84]	3.4%
Zhu et al., 2011	73	−13.50	19.96	73	−0.40	18.20	−0.68	[−1.02; −0.35]	5.6%
Basu et al., 2011	15	−5.00	26.03	16	4.00	26.22	−0.34	[−1.05; 0.37]	2.5%
Hassellund et al., 2013	27	3.19	0.78	27	3.09	0.83	0.12	[−0.41; 0.66]	3.6%
Zhu et al., 2013	73	−0.35	0.52	73	0.01	0.50	−0.70	[−1.04; −0.37]	5.6%
Wright et al., 2013	8	0.00	0.56	8	0.00	0.85	0.00	[−0.98; 0.98]	1.5%
Soltani et al., 2014	25	−11.44	3.28	25	3.28	16.04	−1.25	[−1.86; −0.64]	3.1%
Basu et al., 2014(L)	15	0.30	3.87	15	−1.50	7.36	0.30	[−0.42; 1.02]	2.4%
Basu et al., 2014(H)	15	−2.50	9.30	15	1.70	12.39	−0.37	[−1.10; 0.35]	2.4%
Li et al., 2015	29	−0.25	2.55	29	0.02	2.44	−0.11	[−0.62; 0.41]	3.8%
Zhang et al., 2015	37	0.16	0.56	37	0.27	0.84	−0.15	[−0.61; 0.30]	4.3%
Lee et al., 2016	32	−24.03	30.03	31	−9.75	31.20	−0.46	[−0.96; 0.04]	3.9%
Yang et al., 2017	80	−0.29	0.82	80	−0.10	0.84	−0.23	[−0.54; 0.08]	5.9%
Xie et al., 2017	25	−18.50	39.00	24	−0.40	28.90	−0.52	[−1.09; 0.05]	3.3%
Bakuradze et al., 2019	30	12.30	26.64	27	19.60	30.45	−0.25	[−0.77; 0.27]	3.7%
Guo et al., 2020(M)	18	0.06	0.50	15	0.07	0.51	−0.02	[−0.70; 0.67]	2.6%
Guo et al., 2020(VL)	19	0.02	0.48	15	0.07	0.51	−0.10	[−0.78; 0.58]	2.7%
Guo et al., 2020(L)	19	−0.01	0.42	15	0.07	0.51	−0.17	[−0.85; 0.51]	2.7%
Guo et al., 2020(H)	17	0.16	0.51	15	0.07	0.51	0.17	[−0.52; 0.87]	2.6%
Guo et al., 2020(VH)	19	0.04	0.69	15	0.07	0.51	−0.05	[−0.72; 0.63]	2.7%
Xu et al., 2021(M)	42	−0.11	0.45	46	−0.07	0.49	−0.08	[−0.50; 0.33]	4.7%
Xu et al., 2021(L)	45	−0.13	0.81	46	−0.07	0.49	−0.09	[−0.50; 0.32]	4.7%
Xu et al., 2021(H)	43	−0.31	0.47	46	−0.07	0.49	−0.50	[−0.92; −0.07]	4.6%
Aboufarrag et al., 2022(BRE)	52	0.00	0.80	52	−0.10	0.90	0.12	[−0.27; 0.50]	5.0%
Aboufarrag et al., 2022(BE)	52	0.00	0.80	52	−0.10	0.90	0.12	[−0.27; 0.50]	5.0%
**Random effects model**	**912**			**899**			**−0.23**	**[−0.37; −0.10]**	**100.0%**

Heterogeneity: $I^2 = 45\%$, $\tau^2 = 0.0529$, $p < 0.01$

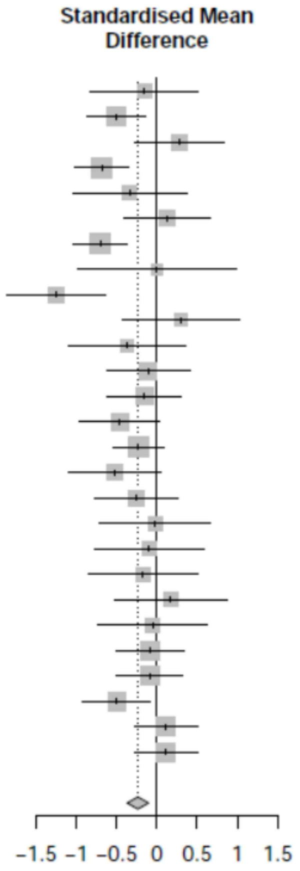

Supplementary Figure 4. Forest plot: low-density lipoprotein-cholesterol

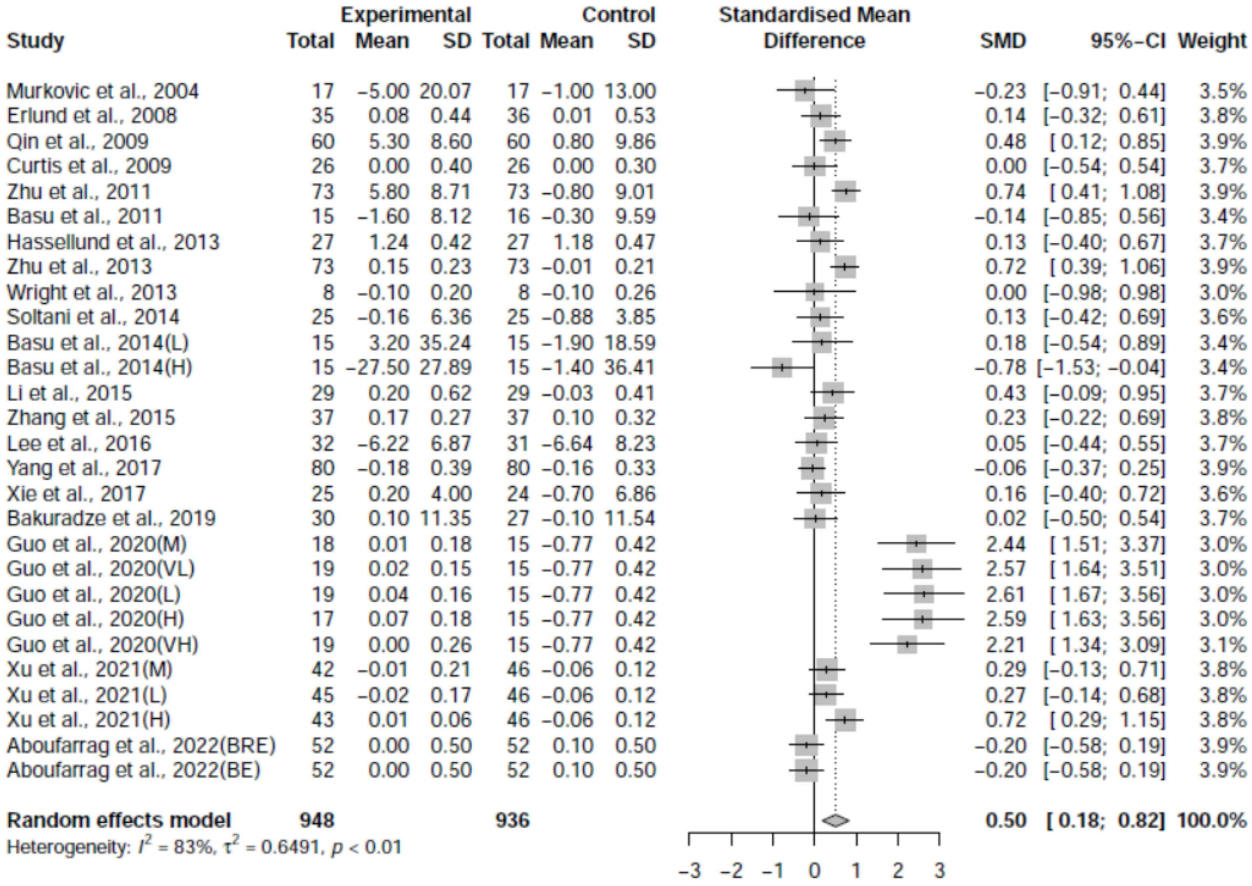

Supplementary Figure 5. Forest plot: high-density lipoprotein-cholesterol

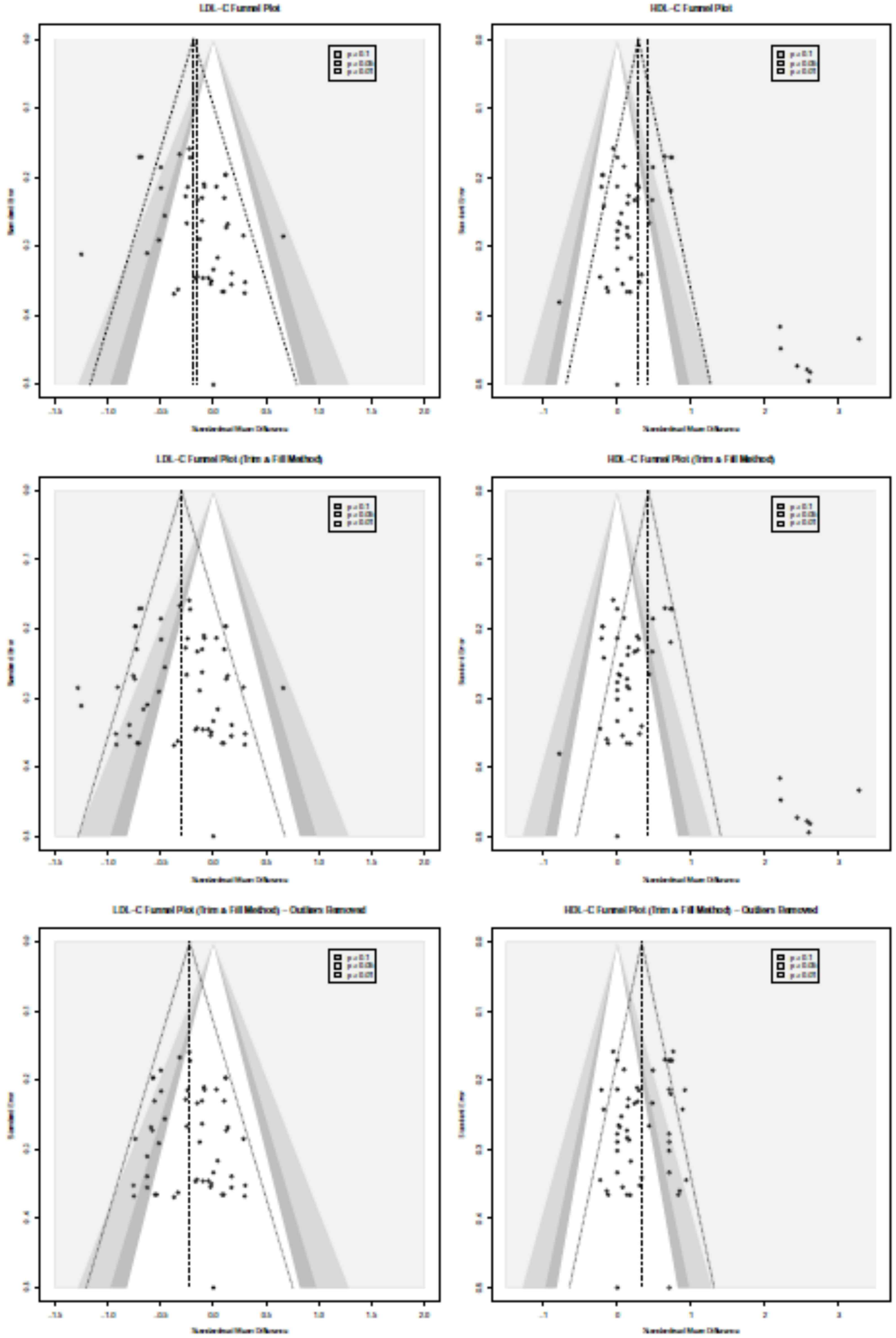

Supplementary Figure 6. Funnel plot for publication bias
